# Snow and Glacial Algae: A Review^1^

**DOI:** 10.1111/jpy.12952

**Published:** 2020-02-29

**Authors:** Ronald W. Hoham, Daniel Remias

**Affiliations:** ^1^ Department of Biology Colgate University Hamilton New York 13346 USA; ^2^ School of Engineering University of Applied Sciences Upper Austria Wels 4600 Austria

**Keywords:** albedo, community structure, cryophilic, environmental parameters, genomics, glacial algae, life cycles, primary productivity, secondary metabolites, snow algae

## Abstract

Snow or glacial algae are found on all continents, and most species are in the Chlamydomonadales (Chlorophyta) and Zygnematales (Streptophyta). Other algal groups include euglenoids, cryptomonads, chrysophytes, dinoflagellates, and cyanobacteria. They may live under extreme conditions of temperatures near 0°C, high irradiance levels in open exposures, low irradiance levels under tree canopies or deep in snow, acidic pH, low conductivity, and desiccation after snow melt. These primary producers may color snow green, golden‐brown, red, pink, orange, or purple‐grey, and they are part of communities that include other eukaryotes, bacteria, archaea, viruses, and fungi. They are an important component of the global biosphere and carbon and water cycles. Life cycles in the *Chlamydomonas–Chloromonas–Chlainomonas* complex include migration of flagellates in liquid water and formation of resistant cysts, many of which were identified previously as other algae. Species differentiation has been updated through the use of metagenomics, lipidomics, high‐throughput sequencing (HTS), multi‐gene analysis, and ITS. Secondary metabolites (astaxanthin in snow algae and purpurogallin in glacial algae) protect chloroplasts and nuclei from damaging PAR and UV, and ice binding proteins (IBPs) and polyunsaturated fatty acids (PUFAs) reduce cell damage in subfreezing temperatures. Molecular phylogenies reveal that snow algae in the *Chlamydomonas–Chloromonas* complex have invaded the snow habitat at least twice, and some species are polyphyletic. Snow and glacial algae reduce albedo, accelerate the melt of snowpacks and glaciers, and are used to monitor climate change. Selected strains of these algae have potential for producing food or fuel products.

**Abbreviations**
*Cr*: *Chloromonas*
*Cd*: *Chlamydomonas*
*Ch*: *Chlainomonas*
DIC: dissolved inorganic carbon
DOC: dissolved organic carbon
DON: dissolved organic nitrogen
HTS: high‐throughput sequencing
IBPs: ice binding proteins
MAAs: mycosporine‐like amino acids
OTUs: operational taxonomic units
PUFAs: polyunsaturated fatty acids
TXT: triple crossover triangle

Snow may cover up to 32% of the Earth's land surface and ice up to 11% (Allison et al. 2018). This review on algae that live in these habitats is an update since Hoham and Duval (2001) in the comprehensive reference on snow ecology (Jones et al. 2001), and we use the original taxonomic names used by authors in their papers even though changes have been made since then. Previous overviews in this area of phycology include those of Kol (1968), Hoham (1980), and Hoham and Ling (2000). Additional reports on cell structure and physiology (Remias 2012), adaptation strategies (Leya 2013), ecology, systematics, and life cycles (Komárek and Nedbalová 2007), glacial ice algae (Williamson et al. 2019), glacial ecosystems (Hodson et al. 2008), and cold alpine regions (Sattler et al. 2012) have further contributed to our understanding of these algae**.** Organisms regarded as true snow and glacial algae thrive in a liquid water film between melting snow and ice crystals, and usually do not propagate outside of this habitat. Otherwise, microalgae of different origins such as bare soils and from lichen fragments may be passively transported onto snow and ice surfaces by meltwater inflow or wind. Under certain conditions, they may even cause a snow discoloration, but are not regarded as true snow or glacial algae in the strict sense. Microbial communities that inhabit snow and glacial ice are not only abundant and taxonomically diverse and complex in terms of their interactions, but their role in global biogeochemical cycles has been underestimated (Maccario et al. 2015, Havig and Hamilton 2019, Williamson et al. 2019). Algal blooms typically occur from one to several weeks during spring and summer when air temperatures remain above 0°C in semi‐permanent snowfields and glaciers in temperate, mountainous, and polar regions (Hoham and Duval 2001). The most prominent snow algae belong to the Chlamydomonadales (Chlorophyta) and glacial algae to the Zygnematales (Streptophyta). Yet, other groups of algae may color snow including euglenoids, cryptomonads, chrysophytes, and dinoflagellates (Hoham and Duval 2001). When the concentration of cells reaches a population in several thousands of cells mL^−1^, a snow or ice discoloration takes place. The color and its intensity depend on the pigment composition and population density. When chlorophylls dominate, green snow appears (Chlamydomonadales; Figs. 1a and 2a). If primary carotenoids like fucoxanthin dominate, golden‐brown snow appears (Chrysophyceae; Figs. 1b and 2b). In many cases, the pigment composition can vary depending on the stage of the life cycle. Most prominent are secondary carotenoids like astaxanthin of certain chlamydomonadalean green algae, which dominate over chlorophylls to cause orange (Figs. 1c and 2c), pink (Figs. 1d and 2d), and red snow (Figs. 1e and 2e). Purple to brown phenols abundantly present in glacial streptophytic algae (Zygnematales) cause grey snow or purple ice (Figs. 1f and 2f); however, this color is frequently masked by dark cryoconite particles which are common at surfaces of old ice.

**Figure 1:**
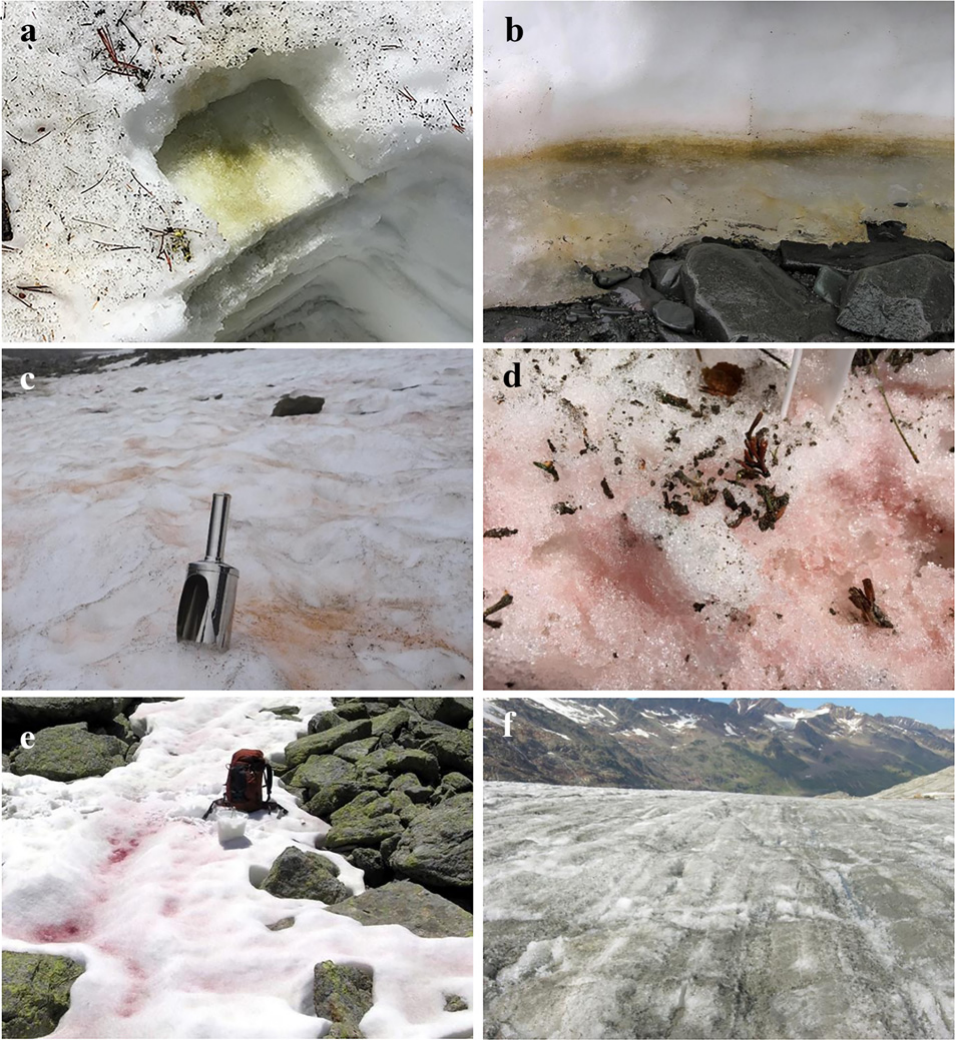
Field images of snow and glacial algae. (a) Green snow, *Chloromonas brevispina* (Chlorophyta, Chlamydomonadales), Carson Mountains, NV, June 2016. (b) Golden‐brown snow, *Hydrurus* sp. (Chrysophyceae), King George Island, Antarctica, January 2009. (c) Orange snow, *Sanguina aurantia* (Chlorophyta, Chlamydomonadales), Svalbard (Norway), July 2018. (d) Pink snow, *Chlainomonas kolii* (Chlorophyta, Chlamydomonadales), Donner Pass, CA, June 2016. (e) Red snow, *Sanguina nivaloides* (Chlorophyta, Chlamydomonadales), European Alps, Austria, July 2008. (f) Grey‐colored glacier, *Mesotaenium berggrenii* (Streptophyta, Zygnematales), Gurgler Glacier, Austria, August 2017.

**Figure 2:**
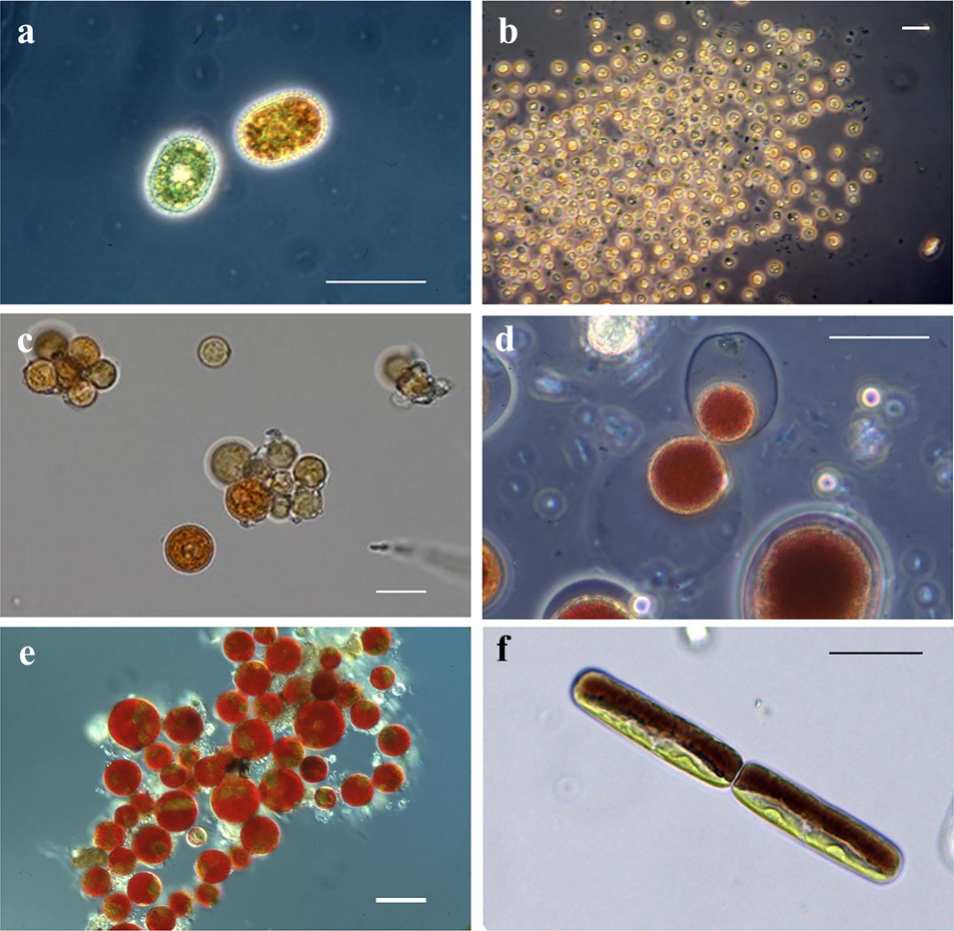
Photomicrographs (Nomarski‐interference and phase‐contrast) of snow and glacial algae that correspond to field images in Figure 1 except for b, d, e, and f noted below. (a) Green and orange zygotes of *Chloromonas brevispina*. (b) Golden‐brown vegetative cells of *Chromulina chionophilia* (Chrysophyceae; Pugh Mtn., WA; photomicro*graphs of *Hydrurus* sp. were not available). (c) Orange cysts of *Sanguina aurantia*. (d) Red vegetative cell of *Chlainomonas rubra* showing external cell division (see text). (e) Red to green cysts in *Chlamydomonas nivalis*. (f) Purple‐green vegetative cells of *Ancylonema nordenskiöldii* from a grey‐colored glacier (Streptophyta, Zygnematales, Morteratsch Glacier, Switzerland). Scale bars = 20 μm for a, c, d, and e and 10 μm for b and f.

Molecular phylogenies have enhanced our understanding of the evolutionary history of snow species in the *Chlamydomonas–Chloromonas–Chlainomonas* complex (Hoham et al. 2002, 2006, Novis et al. 2008, Muramoto et al. 2010, Remias et al. 2013b, 2016, 2018, Matsuzaki et al. 2014, 2015, 2018, 2019, Procházková et al. 2019a,b). Metagenomics (Hisakawa et al. 2015, Lutz et al. 2015a, Raymond 2016, Maccario et al. 2019), lipidomics (Řezanka et al. 2014), and HTS (Lutz et al. 2015a, 2018, 2019, Segawa et al. 2018) have significantly contributed new information. Samples from difficult inaccessible regions and satellite imaging of large ice sheets showing algal abundance or melting processes has widened our knowledge about biodiversity and occurrence (Takeuchi et al. 2006a, Hisakawa et al. 2015). In an Alaskan 1,900 km^2^ icefield, red snow extended over 700 km^2^ and microbial communities were responsible for 17% of the snow melt (Ganey et al. 2017). Their contribution to climate warming is likely to grow with increased melt and nutrient input. Laboratory studies have furthered our comprehension of difficult life cycles (Hoham et al. 2006), pH optima (Hoham et al. 2007), temperature optima (Hoham et al. 2008a), effects of coniferous leachates on growth (Hoham et al. 2008b), and interactions of light on growth and life cycle development (Hoham et al. 2000a,b, 2009). Snow and glacial algae are examples of how life can adapt to harsh environmental conditions in terms of solar irradiance, low temperatures or nutrients, and show that phototroph extremophiles perform well in putative extreme habitats such as melting snowpacks or glacial surfaces. As a result, these microbes have been considered as Earth analogs for life outside our planet (Havig and Hamilton 2019, Vimercati et al. 2019b).

## Diversity and community structure

Snow algae have been found on every continent and are a global phenomenon. Their distributions are limited to suitable habitats depending on snow or permanent ice, ecological, and climatic conditions. Since 2000, they have been recorded from all continents and geographic regions (Table 1) except Australia where they were reported previously from the Snowy Mountains (Marchant 1982). Communities of snow algae are diverse and taxonomically broad, comprised of clones with discrete patches, and are heterogeneous (Brown et al. 2016). Even though they color the snow red, green, orange, or golden‐brown, they still may be an important component of snow even in the absence of these colors (Brown and Jumpponen 2019). Green snow in the Laurentian Mountains, Quebec, was defined as having more than 4,000 cells · mL^−1^, whereas white snow had populations below that level (Hoham and Duval 2001).

**Table 1:** Countries or regions where snow and glacial algae have been found since 2000

Country or Geographic Region	References
Antarctica	Ling (2001), Fujii et al. (2010), Remias et al. (2013a,b), Davey et al. (2019), Procházková et al. (2019a)
Argentina	Procházková et al. (2019a)
Austria	Remias et al. (2005, 2009, 2010a,b, 2012b, 2013b, 2016, 2018, 2019), Holzinger et al. (2016), Procházková et al. (2018a, 2019a)
Bulgaria	Lukavský et al. (2009), Lukavský and Cepák (2010), Cepák and Lukavský (2013)
Canada	Duval and Hoham (2000), Moestrup et al. (2018)
Chile	Takeuchi and Kohshima (2004), Vimercati et al. (2019b)
China	Takeuchi et al. (2008)
Czech Republic	Nedbalová et al. (2008), Řezanka et al. (2008b), Procházková et al. (2019b)
Ecuador	Nedbalová and Sklenár (2008)
Greece	Cepák et al. (2016)
Greenland	Uetake et al. (2010), Yallop et al. (2012), Takeuchi et al. (2014), Stibal et al. (2017), Lutz et al. (2018), Onuma et al. (2018), Wang et al. (2018)
Iceland	Lutz et al. (2015a)
Italy	Procházková et al. (2019a)
Japan	Muramoto et al. (2010), Tanabe et al. (2011), Matsuzaki et al. (2014), Terashima et al. (2017), Matsuzaki et al. (2018, 2019)
New Zealand	Novis (2002a,b), Novis et al. (2008)
Nepal	Takeuchi et al. (2009)
Norway Poland	Procházková et al. (2019a)Procházková et al. (2019b)
Russia	Uetake et al. (2006), Takeuchi et al. (2006b), Hisakawa et al. (2015), Takeuchi et al. (2015), Tanaka et al. (2016), Novakovskaya et al. (2018)
Slovakia	Hanzelová et al. (2018), Procházková et al. (2018a,b, 2019a,b)
Slovenia	Procházková et al. (2019a)
Spain	Cepák and Lukavský (2012)
Svalbard (Norway)	Müller et al. (2001), Stibal et al. (2007), Remias et al. (2009), Kvíderová (2012), Remias et al. (2012a, 2013a), Lutz et al. (2015b, 2017), Barcyt≐ et al. (2018), Procházková et al. (2019a), Takeuchi et al. (2019)
Sweden	Lutz et al. (2017)
Switzerland Tanzania‐Kenya	Procházková et al. (2019a)Vimercati et al. (2019a)
Uganda	Uetake et al. (2014)
USA	Duval and Hoham (2000), Gorton et al. (2001), Gorton and Vogelmann (2003), Hoham et al. (2006), Takeuchi et al. (2006a), Novis et al. (2008), Takeuchi (2009, 2013), Procházková et al. (2019a)

When using HTS to evaluate snowfields such as those dominated by green algae, HTS outputs need to be thoroughly checked when organisms are poorly represented in databases, which is the case for cryoflora (Lutz et al. 2019). An optimized workflow was recommended to include a consistent sampling, a two‐molecular marker approach, light microscopy‐based guidance, generations of appropriate reference sequences, and final manual verification of taxonomic assignments. HTS and subsequent oligotyping on the Greenland Ice Sheet showed an extremely low algal diversity of the streptophytes, *Ancylonema nordenskiöldii* and *Mesotaenium berggrenii* that dominated at all sites (Lutz et al. 2018). Green snow represented a wet, carbon, and nutrient‐rich environment dominated by *Microglena*, whereas red snow was dry, nutrient poor, and colonized by *Chloromonas* (Lutz et al. 2015b). Population densities of *Chloromonas reticulata* reached 0.33 × 10^4^ cells · mL^−1^ in red snow from the Ural Mtns., Russia (Novakovskaya et al. 2018). They found alliance of the Russian strain with other strains of *Cr. reticulata* using ITS2, morphology, and TEM. Golden‐brown snow caused by the chrysophyte, *Hydrurus*, populated water‐logged snow fields in Antarctica and Svalbard (Remias et al. 2013a), and *A*.* nordenskiöldii*, cyanobacteria, and diverse green algae dominated on three different glaciers in Svalbard (Takeuchi et al. 2019). Similarities between specific habitats across glaciers and ice sheets worldwide occur regarding their main primary producers (Anesio et al. 2017). At the surface, cyanobacteria dominate the carbon production in aquatic and sediment systems such as cryoconite holes, while Zygnematales and Chlamydomonadales dominate ice surfaces and snow dynamics, respectively. *Mesotaenium berggrenii* and *A. nordenskiöldii* dominated the ice area on the Akkem Glacier in the Russian Altai Mtns as reported for other glaciers in the Northern Hemisphere, whereas a *Chloromonas* sp. causing a red coloration dominated the snow area (Takeuchi et al. 2006a). *Mesotaenium berggrenii*,* Cylindrocystis brébissonii, Ancylonema* sp., and the desmid *Closterium* sp. dominated the lower elevation of Tyndall Glacier, Chile, *Chloromonas* sp. and an Oscillatoriacean cyanobacterium the middle part, and an unknown alga the upper part (Takeuchi and Kohshima 2004).

Using satellite imagery, distribution of red snow (*Chlamydomonas nivalis*) on Harding Icefield, Alaska, matched field observations with more algae on the continental than the maritime side of the icefield (Takeuchi et al. 2006a). Mean carbon content from the red algal biomass averaged 1.2 kg · km^−2^. Satellite imagery indicated that Qaanaaq Glacier in northwest Greenland had a dark‐colored surface compared to the lighter one on Russel Glacier in west central Greenland due to the former dominated by green algae and the latter by cyanobacteria (Uetake et al. 2010). The biovolume was 2.35 times higher on Qaanaaq Glacier at the same altitude. With Sentinel‐3 imagery, the spatial pattern of glacial algae, *Ancylonema nordenskiöldii* and *Mesotaenium berggrenii*, in Greenland using the reflectance ratios between 709 nm and 673 nm bands was highly consistent with field measurements (Wang et al. 2018). Their analysis revealed widespread proliferation of algae on bare ice from late July to mid‐August with increasing algal populations after the peak of surface runoff and meltwater production.

The only record where filamentous cyanobacteria dominated was from Miaoergou Glaciers in the Kalik Mtns in western China, and no green algae were found, which was common in the northern Tibetan Plateau (Takeuchi et al. 2008). The snow algal community on Rikha‐Samba Glacier in western Nepal consisted of the streptophytes, *Mesotaenium berggrenii* and *Cylindrocystis brébissonii*, and filamentous and coccoid cyanobacteria (Takeuchi et al. 2009). Populations of *Chlamydomonas nivalis* on Gulkana Glacier, Alaska Range, Alaska, dominated on the snow surface and *Ancylonema nordenskiöldii* and *M. berggrenii* on the ice surface in September, and these algae contribute to the net production of organic carbon (Takeuchi 2013). Snow and ice algal communities on glaciers in the Suntar‐Khayata Mtn Range in Russian Siberia were dominated by *A. nordenskiöldii* in the lower bare ice area and *Chloromonas* sp. in the upper snow area (Tanaka et al. 2016). The total algal bio volume showed altitudinal variation ranging from 0.03 to 4.0 mL · m^−2^, was highest in the middle of the glaciers, and was similar on all glaciers. Over 3 years, there was no significant change in community structure, but there was in the total biomass. Pinnacle‐shaped ice structures (“nieves penitentes”) in high elevations (5,277 m a.s.l.) of the dry Chilean Andes supported red ice patches dominated by *Chlamydomonas* and *Chloromonas*, which were closely related to snow algae from alpine and polar regions (Vimercati et al. 2019a). These pinnacles provide water and shelter from high winds, high UV irradiance, and thermal fluctuations in this otherwise extreme landscape that was suggested as a terrestrial analog for astrobiological studies for life outside Earth.

Populations of snow and glacial algae, food chains, food webs, and associated physical and chemical parameters make up complex snow and glacial ecosystems (Aitchison 2001, Hoham and Duval 2001). Bacteria, fungi, archaea, and algae are normal inhabitants of glacial surfaces (Lutz et al. 2015a, Ciccazzo et al. 2016) that may also include viruses and metazoans (Sattler et al. 2010). Green algae, cyanobacteria, bacteria, fungi, and pollen on the Sofiyskiy Glacier in the Altai Mtns Russia were used to date ice cores that contained 16 annual layers marking summer layers when present and winter layers when absent (Uetake et al. 2006). Using a HTS approach for microbial communities on Icelandic glaciers, snow algae (*Chloromonas polyptera*,* Raphidonema sempervirens*, and two chlamydomonads) were detected supporting a community of other microbes (eukaryotes, prokaryotes, archaea; Lutz et al. 2015a). Employing 18S rRNA, several species of green algae, fungi, and various bacterial phylotypes were detected from red snow in Langhovde, Antarctica (Fujii et al. 2010). The bacteria found were closely related to psychrophilic heterotrophic strains with *Hymenobacter* being the most prominent. The site was enriched with ^15^N and the primary source was fecal pellets from seabirds. From green and red‐colored snow on Mt. Asahi, Japan, *Chloromonas* spp. dominated all samples and *Chlamydomonas* was second most abundant in red snow (Terashima et al. 2017). Bacteria from the subphylum Betaproteobacteria were frequent in both green and red snow, while members of the phylum Bacteroidetes were prominent in red snow. Using bacterial 16S rRNA, 13 phyla and 82 genera of bacteria were found on glaciers in the Tibetan Plateau (Yongqin 2011). In red and green snow in Ryder Bay, Antarctic Peninsula, green communities that consisted of *Chloromonas*,* Chlamydomonas*, and *Chlorella* had a high chlorophyll content, and both communities contained bacteria, protists, and fungi (Davey et al. 2019). At several New England, USA ski slopes, orange cysts of an unidentified *Chloromonas* were associated with filamentous fungi, rotifers, and ciliates (Duval and Hoham 2000). These cysts were found only on ski slopes, which exemplifies this habitat needs further investigations. In the Carpathians of Slovakia, a snow community included *Chloromonas nivalis*, cyanobacteria, fungi, ciliates, rotifers, nematodes, and tardigrades (Hanzelová et al. 2018). From glacial ice at the top of Mt. Kilimanjaro on the Tanzania‐Kenya border, bacterial OTUs (16S rRNA) were dominated by Proteobacteria, Bacteroidetes, Actinobacteria, and Acidobacteria (Vimercati et al. 2019b). Cyanobacteria represented 10% of sequences with most in the Oscillatoriales and Chroococcales. Eukaryotic OTUs (18S rRNA) revealed that Chlorophycean green algae comprised about 9% of total ice sequences and were most closely related to *Chlamydomonas, Chloromonas*, and *Stigeoclonium*. Cercozoa dominated the ice communities representing 72% of total sequences. It is believed that under present conditions of climate change, rapid glacial shrinking at the top of Kilimanjaro, which supports both cosmopolitan and endemic microbial communities, will continue unabated and the entire summit of the mountain is expected to be devoid of ice for the first time in 11,000 years by mid‐century. Metagenomic and satellite analyses from Franz Josef Land in the Russian Arctic confirmed that white snow and ice were initially colonized by fungal dominated communities that were replaced with more complex red snow communities of *Cd. nivalis*, which supported complex viral and heterotrophic bacterial communities (Hisakawa et al. 2015). By comparing metagenomes from snow samples collected in a Greenland sea ice snow cover, composition and function of microbial communities were influenced primarily by atmospheric deposition and in flow of sea ice brine that form a snow‐specific assemblage reflecting the particular environmental conditions of the snowpack (Maccario et al. 2019).

Cryoconite holes play important roles in glacial ecosystems (Takeuchi 2011). Rotifers, tardigrades, copepods, and midge larvae are sustained by snow algae and cyanobacteria on Himalayan glaciers, whereas ice worms and collembola are common in North American glaciers. Cryoconite granules from ice areas on northwest Greenland glaciers are aggregates of mineral particles, filamentous cyanobacteria, other microbes, and organic matter, while those in snow areas consisted of mineral particles and snow algae (Takeuchi et al. 2014).

## Life cycles and reproductive strategies

Many snow algae that appear during snow melt come from the germination of underlying resting spores on the ground surface that have been dormant from the end of snow melt the previous year until germination the current year (Hoham and Duval 2001, Hoham et al. 2006). Populations are typically found in the same localities from year to year. However, aerial distribution was documented in the Canadian High Arctic where 47% of the cyanobacterial operational taxonomic units (OTUs) using SSU rDNA were found in microbial mats in the region indicating this group was substantially derived from local sources (Harding et al. 2011). Cysts of *Chlamydomonas nivalis* and *Sanguina nivaloides* may germinate locally in the same areas year after year as mentioned above or be dispersed geographically by strong winds particularly in polar regions (Müller et al. 2001, Procházková et al. 2019a).

**Figure 3:**
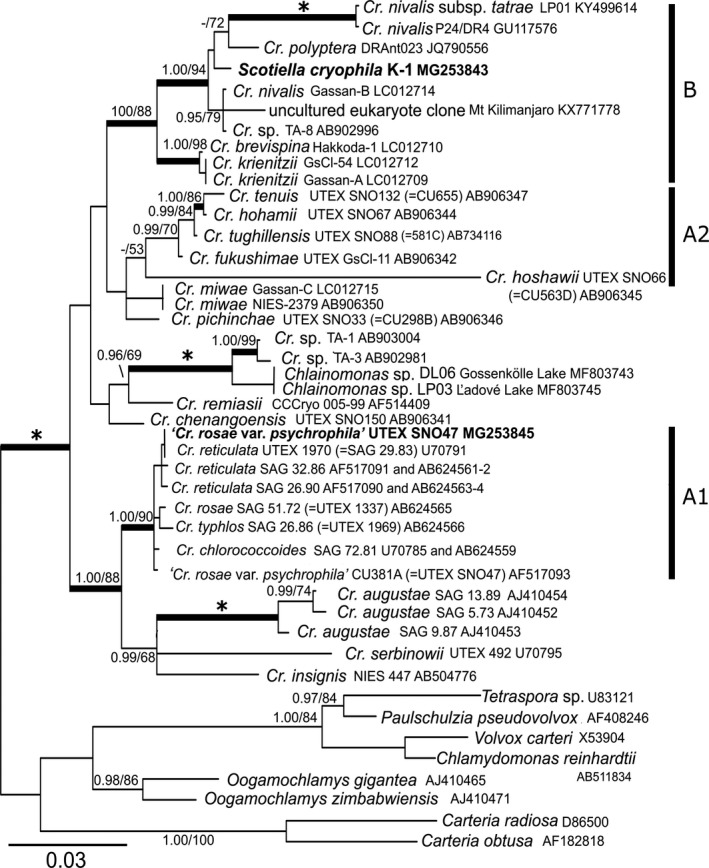
18S ribosomal DNA gene‐based Bayesian phylogenetic tree on *Chloromonas* focusing on snow‐inhabiting and mesophilic relatives. Full statistical support (1.00/100) is marked with an asterisk (from Remias et al. 2018 with permission of Taylor & Francis Group, LLC,* Phycologia*, Philadelphia).

The chlamydomonadalean snow algae *Chloromonas, Chlamydomonas*, and *Chlainomonas* have different reproductive strategies, many of which are unclear. Moreover, the morphology of a species may undergo striking changes from a vegetative flagellate via planozygotes to an immobile cyst or spore stage (Remias 2012). The snow alga, *Chloromonas hindakii*, was found in orange snow in three European mountains ranges, Krkonoše and Jesenίky (Czech Republic) and High Tatras (Poland and Slovakia; Procházková et al. 2019b). Using 18S rDNA, *rbc*L, and ITS2 rDNA phylogenetic analyses and morphological traits from field and cultured material, *Cr. hindakii* was determined to be a new species. Orange cysts associated with the vegetative cells resemble those of *Chloromonas nivalis*, but it is not known if they are produced asexually or sexually. Three species of *Chloromonas* in Upstate New York were studied extensively, each with a different reproductive strategy (Hoham et al. 2002, 2006). *Chloromonas chenangoensis* is homothallic, produces planozygotes, but mature zygotes were not seen. *Chloromonas tughillensis* is heterothallic with MT+ and MT− mating types and produces spherical zygotes that are green to orange (Hoham et al. 2006). The third species, *Cr. rosae* v. *psychrophila*, produces only asexual resting spores directly from biflagellate vegetative cells, and these resting spores were formerly identified as *Scotiella cryophila*. The *Scotiella cryophila* K‐1 cell type from the Austrian Alps is genetically different from *Cr. rosae* v. *psychrophila* from North America (Fig. 3; Remias et al. 2018). Another problem in this group is *Cr. rosae* from the American Southwest that produces planozygotes resembling *Cryocystis granulosa* Kol (Hoham and Blinn 1979), but attempts to produce sexual zygotes in the laboratory did not meet with success (R. Hoham, pers. obs.).

Species of *Chlainomonas* can divide externally forming new protoplasts outside the parent cell unlike other chlamydomonadalean green algae (Hoham 1974b, Novis et al. 2008; Fig. 2D). These protoplasts may form new cells directly or possibly fuse with one another forming thick‐celled resting spores that are either smooth walled or with spine‐like projections (*Chlainomonas rubra*, Hoham 1974b; *Chlainomonas kolii*, Novis et al. 2008). However, vegetative cells of *Chlainomonas* sp. form conventional internal daughter cells (Procházková et al. 2018a) and germination of cysts also produces internal daughter cells in *Ch. rubra* (Hoham 1974b). Phylogenetic analysis using *rbc*L sequences places *Chlainomonas* in the *Chloromonas* clade of snow algae (Novis et al. 2008), and its quadriflagellate condition is hypothesized to be derived unlike other chlorophycean chlamydomonadalean flagellates where it is ancestral. An unidentified species of *Chlainomonas* was found in the Tyrolean Alps, Austria, and resistant cells were spherical with smooth walls (Remias et al. 2016), and this same taxon was found in the High Tatras, Slovakia (Procházková et al. 2018a).

The life cycle of *Chlamydomonas nivalis* remains unclear for several reasons. Blooms of snow algae have a long history of being labeled as *Cd. nivalis* (Kol 1968, Hoham and Duval 2001) with minimal evidence of their affinity to the type collection or other blooms given the same name. Consequently, the name had been so broadly applied that attempts to reconcile the identities of these various collections are likely fruitless. However, Procházková et al. (2019a) have recently shown that globally distributed spherical red cysts of snow algae frequently causing blooms form a taxonomic clade according to 18S rDNA and ITS2 marker sequences, and they were placed in the new genus *Sanguina*. Thus, it is possible that many of the blooms labeled as *Cd. nivalis* in the past, perhaps extending back to the type of the latter species by F. Bauer, represent *Sanguina nivaloides* (or *S. aurantia* if smaller cells and orange cysts). Since this cannot be determined in retrospect, a formal synonymy between *S. nivaloides* and *Cd. nivalis* is impossible.

Comments about the life cycle of *Sanguina* may apply to many collections of *Chlamydomonas nivalis*, but this also cannot be determined due to the above‐mentioned reasons. In field samples, red spherical cells with smooth walls that have been interpreted as asexual resting spores are very abundant in mountainous and polar regions. However, spherical cells with extended processes along the outer wall are rarer and have been interpreted as sexual zygotes even though this has never been documented. The typical red cyst is smooth‐walled, but populations may also consist of cells with extensions, or with smooth papillae extending from the cell wall. The orange cysts of *Sanguina aurantia* may or may not have an outer distant cell layer and differ from the red cysts of *S. nivaloides* in the structure of the second layer of the cell wall being multilayered and slightly undulating on the surface (Procházková et al. 2019a).

In the life cycles of *Chloromonas tughillensis* and *Cr. chenangoensis*, cell shape transitions from oblong to spherical occur over an 8 h period after the onset of light (Hoham et al. 2006). Peaks in cell transitions, total spheres, and total planozygotes differed slightly between these two species. Young cysts of *Cr. nivalis* with one large chloroplast fragmented into several smaller ones as cysts aged and enlarged (Remias et al. 2010b), and the number of chloroplasts per cell varied from one to several in vegetative cells of *Cr. chenangoensis* (Hoham et al. 2006).

Conjugation between two filaments produces oblong zygotes in the streptophyte, *Ancylonema nordenskiöldii*, in glacial ice from Svalbard (Remias et al. 2012a). The dinoflagellate, *Borghiella pascheri*, which causes red snow on frozen lakes in the European Alps and in Ontario, Canada, forms temporary cysts surrounded by fused inner membranes of the amphiesmal vesicles and sexual reproduction produces hypnozygotes (Moestrup et al. 2018).

## Primary productivity and secondary metabolites

Primary productivity was examined from glaciers on stratovolcanoes in the Pacific NW, USA, and most was attributed to photoautotrophic snow algal communities (Hamilton and Havig 2017), and increasing levels of CO_2_ correlated with increased primary productivity in snow algae (Hamilton and Havig 2018). Cyanobacteria on glaciers in Svalbard produce glue‐like extracellular polymeric substances that aggregate organic and inorganic debris (cryoconite) by cementing mineral grains (quartz and dolomite), and these dark‐colored aggregates grow and reside on glacial surfaces for years (Hodson et al. 2010). Coastal snowpacks had greater bacterial diversity and autotrophic biomass on Livingston Island, Antarctica, than snowpacks <1 km from the coast and greater amounts of nutrients from rock debris and marine fauna, higher amounts of DIC and CO_2_ in interstitial air, and a close relationship between chlorophyll and DOC. On the Greenland Ice Sheet, algal cell abundance, which ranged from 90 cells · mL^−1^ to 0.98 × 10^4^ cells · mL^−1^, increased significantly with the amount of visible impurities seen on the ice surface (Holland et al. 2019). Correlations between average algal cell counts and DON and DOC surface ice concentrations were significant. Growth and oxygen evolution capacity in *Chlamydomonas nivalis* were low at 2°C, enhanced at 10°C, and were significantly higher at 5–15°C when compared to the mesophilic *Cd. reinhardtii* (Lukeš et al. 2014). Molecular mechanisms responsible for adaptation to photosynthesis at low temperature were attributed to high rates of Q_A_ to Q_B_ electron transfer. Photosynthetic and respiratory data support the cryophilic adaptation of *Cd. nivalis* in the Austrian Alps, but cells produced oxygen without inhibition at temperatures up to 20°C and maintained this for 1 h at irradiances up to 1,800 μmol PAR · m^−2^ · s^−1^ (Remias et al. 2005). Chlorophyll and primary carotenoid pigment composition was similar to that found in most other Chlorophyta. Photosynthetic relative electron rates measured using a Walz fluorimeter in cysts of *Chloromonas nivalis* from the Austrian Alps at 54–1,394 μmol PAR · m^−2^ · s^−1^ peaked at the highest irradiance level tested (Remias et al. 2010a). Using a modified LiCor gas‐exchange system, CO_2_ uptake to 0.3 μmol PAR. m^−2^. s^−1^ occurred in dense blooms of *Chlamydomonas nivalis* in the Snowy Range of the Rocky Mts, USA (Williams et al. 2003). CO_2_ uptake at 2,300 μmol PAR · m^−2^ · d^−1^ occurred in heavily colonized patches indicating that summer snowfields can be very productive. Red light was more effective for CO_2_ uptake than white and much more than green or blue due to the red astaxanthin that surrounds and masks the algal chloroplasts. Supraglacial cyanobacteria and algae capture CO_2_ from the atmosphere and convert it into organic matter, which is broken down and combined with transported organic matter to generate CO_2_ that is released back into the atmosphere (Stibal et al. 2012). The balance between these two processes will determine if a glacier is a net sink or source of CO_2_. Ice sheet interiors function as sinks and ice sheet edges and small glaciers act as a source. Glacial algal assemblages are a potentially important yet under researched component of the global biosphere and carbon and water cycles (Havig and Hamilton 2019, Williamson et al. 2019).

Astaxanthin linked with two glucoses and fatty acids were identified in *Chlamydomonas nivalis* as a diglucoside diester using HPLC and Mass Spectrometry (Řezanka et al. 2008b) and *trans*‐ and *cis*‐forms of astaxanthin accumulate in this species of which the latter additionally absorbs in the UV with a shoulder peaking at 375 nm (Remias and Lütz 2007). Astaxanthin derivatives in *Chlamydomonas nivalis* from the Austrian Alps, Slovak High Tatra Mtns, and Bulgaria Pirin using HPLC showed differences in their composition of optical isomers with a dominance of diglucoside diesters from the Pirin Mtns (Řezanka et al. 2013). Carotenoids and phenols (astaxanthin in snow algae and purpurogallin in glacial algae) shield the photosynthetic apparatus by absorbing visible wavelengths dissipating the excess radiant energy as heat (Dial et al. 2018). This heat is thought to melt proximal ice crystals providing liquid water at 0°C freeing up nutrients bound in frozen water. They also hypothesized that green colored snow algae occupy saturated snow where water is not limiting, and red colored snow algae occupy drier more water limited snow. The accumulation of secondary carotenoids and a decline of chlorophyll in *Chlamydomonas* red snow was attributed to breakdown into phaeophytin caused by UV radiation using Raman spectroscopy at exposed surfaces on McLeod Glacier, Signy Island, Antarctica (Edwards et al. 2004). Raman spectroscopy was also used in the Krkonoše Mts., Czech Republic and Ötztal Alps, Austria, to detect astaxanthin in different stages in the life cycles of the snow algae, *Chloromonas nivalis* and *Chlamydomonas nivalis* (Jehlička et al. 2016). Green communities of *Chloromonas*,* Chlamydomonas*, and *Chlorella* from Ryder Bay, Antarctic Peninsula were protein rich and contained metabolites associated with nitrogen and amino acid metabolism (Davey et al. 2019). Red communities of *Chloromonas* had high carotenoid content and contained more metabolites associated with carbohydrate and fatty acid metabolism. MAAs also offer protection from UV and were found in higher concentration in green snow of unknown species composition than in red snow from King George Island, Antarctica (Kim et al. 2018). Generally, chlamydomonadalean and streptophytic snow and glacial algae do not accumulate MAAs (D. Remias, pers. obs.). Two chrysophytes from golden‐brown snow in Japan, *Ochromonas smithii* and *O. itoi*, use only the violaxanthin cycle for photoprotection as a dissipation system of surplus energy under prolonged high light stress as determined with a PAM chlorophyll fluorimeter (Tanabe et al. 2011).

Many microorganisms survive in cold environments by secreting IBPs that control growth and ice around them. An IBP (*ChloroIBP*) was identified and characterized in a freshwater *Chloromonas* sp. from Antarctica (Jung et al. 2016). Four isoforms of an extracellular IBP in a *Chlamydomonas* from Antarctica did not resemble any known antifreezes, had strong recrystallization inhibition activity, and had the ability to slow the drainage of brine from sea ice (Raymond et al. 2009). These properties, by maintaining liquid environments, may increase survival of cells in freezing temperatures. The IBPs had a repeating TXT motif, which has previously been implicated in ice binding in insect antifreezes and a ryegrass antifreeze. In the snow alga, *Chloromonas brevispina*, genes were found for over 20 IBP isoforms all of which matched fungal and bacterial proteins than algal IBPs providing evidence that the genes were acquired by horizontal transfer (Raymond 2014). However, it remains unclear as to what extent snow algae can export proteins in the nitrogen‐poor environment in which they mostly live.

PUFAs formed nearly 50% of total lipids dominated by phospholipids and glycolipids that would select for life at or near the freezing point in *Chloromonas nivalis* v. *tatrae* from the High Tatra Mtns, Slovakia (Procházková et al. 2018b). Cysts of the snow alga, *Chloromonas hindakii*, from mountain ranges in the Czech Republic, Poland, and Slovakia showed high levels of PUFAs (65.8% and 58.1% of total lipids), whereas the content of saturated acids did not exceed 23% (mainly palmitic acid, 16:0; Procházková et al. 2019b). This combination was regarded as an adaptation to cold temperatures. PUFAs identified by gas chromatography‐mass spectrometry (GC‐MS) made up more than 75% of total fatty acids in *Chloromonas brevispina* from the Bohemian Forest, Czech Republic (Řezanka et al. 2008a). Also, *Chlainomonas* sp. from ice covers of European mountain lakes abundantly accumulates PUFAs (Procházková et al. 2018a). Using lipidomics, snow species in *Chloromonas* were found to be a major producer of C16 PUFAs (16:3 and 16:4; Řezanka et al. 2014).

Vegetative cells of the glacial ice streptophyte, *Mesotaenium berggrenii*, from the Austrian Alps produce high amounts of intraplastidial starch, cytoplasmic lipid bodies, and numerous peripheral vacuoles that housed an unidentified brownish pigment that screened for irradiance damage (Remias et al. 2009). This pigment was subsequently identified as a UV and VIS absorbing purpurogallin derivative, a unique phenolic compound to this group of algae (Remias et al. 2012b). Similarly, *Ancylonema nordenskiöldii*, from glacial ice in Svalbard had peripheral brownish vacuoles that absorbed potentially damaging PAR and UV (Remias et al. 2012a). Photosynthesis measurements at 1°C and different light levels in *M. berggrenii*, and different temperatures and light levels in *A. nordenskiöldii*, indicated that metabolism is adapted to temperatures near freezing and to high light conditions in both species. From 48 to 1362 μmol PAR · m^−2^ · s^−1^ oxygen production continued to rise with no inhibition at the highest level in cysts of *Chloromonas polyptera* from Antarctica (Remias et al. 2013b).

## Genomics, systematics, and evolution

The rise of molecular methods brought many new insights to the systematics of snow algae in the last two decades. The molecular phylogeny using SSU rRNA and taxonomic revision of the *Chlamydomonas–Chloromonas* complex in the chlamydomonadalean green algae revealed seven different clades confirming the polyphyly of the two genera (Pröschold et al. 2001). However, *Chloromonas* was emended on the basis of chloroplast characters and all species with or without pyrenoids were placed in a monophyletic clade with the type species, *Chloromonas reticulata*. In contrast, *Chlamydomonas* is a polyphyletic genus and the main cause of red snow traditionally associated with *Chlamydomonas nivalis*, which was recently re‐described creating a new genus *Sanguina* with two species, *S. nivaloides* and *S. aurantia* (Procházková et al. 2019a). They showed that *S. nivaloides* is a diverse cosmopolitan species with 18 haplotypes using ITS2 rDNA analysis with low nucleotide divergence (≤3.5%). Though they did not attempt to synonymize *Cd. nivalis* with *S. nivaloides* because there is no conclusive way to do so, for completeness, an overview of the nomenclatural complexity related to *Cd. nivalis* was given.

The first comprehensive molecular phylogeny of *Chlamydomonas–Chloromonas* snow species used 18S rDNA and *rbc*L gene sequence analysis (Hoham et al. 2002). The 21 cold‐tolerant taxa of which 10 were from snow, occurred in four distinct clades, suggested at least five origins in cold habitats, and all snow species occurred in a single clade. The snow species occurred in two groups in subclade 1 and a third group was in subclade 2, which suggested that the snow habitat had been colonized at least twice and possibly three times in its evolutionary history. This phylogeny supported previous findings that pyrenoids have been gained and lost several times within this complex. In all, 48 species of green algae were recognized from snow of which 18 were in the *Chlamydomonas–Chloromonas* complex (Komárek and Nedbalová 2007) and 15 species of *Chloromonas* from snow and ice were separated in a taxonomic key using cytological and morphological features of which 10 were examined using cultured material (Matsuzaki et al. 2015).

Bipolar phylotypes accounted for 37.3% of all sequences using 18S rRNA and ITS2 sequences from Arctic and Antarctic red snow samples, suggesting that red snow algal blooms in polar regions may comprise cosmopolitan phylotypes but also include endemic species (Segawa et al. 2018). Six snow‐inhabiting species of *Chloromonas* with elongate or ellipsoidal vegetative cells were examined using LM and TEM for differences in vegetative cell shape, chloroplast morphology, the number of zoospores within the parental cell, and the formation of cell aggregates in old cultures (Matsuzaki et al. 2014). Their multigene analysis along with ITS2 rDNA separated four of the six species within a small clade *(Chloromonas fukushimae, Cr. hohamii, Cr. tenuis, Cr. tughillensis*), and all six species including *Cr. chenangoensis* and *Cr. pichinchae* were in a clade of snow algae. Using *rbc*L and 18S rRNA analyses, a new species isolated from snow in Svalbard, *Cr. arctica*, nested within a clade containing a number of psychrotolerant strains in the Chloromonadinia phylogroup (Barcyt≐ et al. 2018). The ITS2 rDNA marker showed support for a new species differing from its closest matches, *Chlamydomonas gerlofii* and *Cr. reticulata*, by three and five compensatory base changes, respectively. *Chloromonas nivalis* from the Austrian Alps and Svalbard grouped in one clade using 18S rDNA, but separated from *Cr. nivalis* from North America that appeared in a different snow clade, which supported that *Cr. nivalis* is polyphyletic (Remias et al. 2010a). Using LM and multiple gene analyses, zygotes of *Cr. nivalis* and *Cr. brevispina* are polyphyletic and those of *Cr. nivalis* from Japan and Austria represent at least four different lineages all of which are separated from North American strains (Matsuzaki et al. 2015). Zygotes of *Cr. brevispina* from Japan were transferred to a new species, *Cr. krienitzii*, because vegetative cells of *Cr. krienitzii* are different from those of *Cr. brevispina* in North America. Further studies of *Cr. nivalis* isolates from North America and Svalbard using LM., TEM, and multiple gene analyses, yielded two new species, *Cr. hoshawii* and *Cr. remiasii*, and verified that *Cr. nivalis* from North America and Svalbard was separated phylogenetically from zygotes of *Cr. nivalis* from Austria and Japan (Matsuzaki et al. 2018). Similar multiple gene analyses from Japan showed that one lineage of *Cr. nivalis* zygotes belonged to the snow alga, *Chloromonas miwae*, and that a new snow algal species, *Cr. muramotoi*, is sister to *Cr. miwae* (Matsuzaki et al. 2019). *Chloromonas rosae* v. *psychrophila* from North America produces asexual resting spores identical to *Scotiella cryophila* (Hoham et al. 2002). In the Austrian Alps, *S. cryophila* resting spores were found but not the vegetative cells that produce them (Remias et al. 2018). Using 18S rDNA, *rbc*L, and ITS2 rDNA, these European *S cryophila* K‐1 cell types are related to snow species of *Chloromonas*, but form an independent lineage from the North American populations of *Cr. rosae* v*. psychrophila* (Fig. 3). This may be another example of a polyphyletic *Chloromonas* snow species, but until the vegetative cells from the European Alps are sequenced this cannot be resolved. In the Austrian Alps, a new species of chrysophytes, *Kremastochrysopsis austriaca*, colors the snow golden‐brown (Remias et al. 2019). Using 18S rRNA and *rbc*L analyses, this species showed no close phylogenetic relationships to other psychrophilic chrysophytes (*Chromulina chionophilia*,* Hydrurus* sp., or *Ochromonas* spp.).

Several psychrophilic strains of *Chlamydomonas* isolated from snow and ice fields in polar regions (Antarctica, Svalbard, Japan, and Alaska) belong to the green alga, *Microglena* (Demchenko et al. 2012). Using SSU and ITS1 and ITS2 rDNA, phylogenetic analyses revealed that all strains of *Microglena* form a monophyletic lineage within the Chlorophyceae regardless of habitat. However, the polar species form a subclade within the monophyletic lineage.

Transcriptomes as part of the 1000 Genome Project for Plants were done for *Chloromonas rosae* v. *psychrophila* UTEX SNO 47 (AJUW) and *Cr. tughillensis* UTEX SNO 88 (UTRE) listed under Green Algae Chlamydomonadaceae ( http://www.onekp.com/samples/list.php). The metagenome for *Cr. brevispina* UTEX SNO 96 (SRX1114535) is available (Raymond 2016, https://www.ncbi.nlm.nih.gov/sra/SRX1114535[accn]). The metagenome for *Kremastochrysopsis austriaca* (Raymond and Remias 2019) and transcriptome for *Chromulina chionophilia,* CCMP 261 (K. Terpis, pers. comm.) have been completed for these two snow algal chrysophytes.

## Environmental parameters

### Light

Spectral albedo has a remarkable contrast between red snow caused by algae and cryoconite‐covered ice surfaces on Qaanaaq Glacier in northwest Greenland (Aoki et al. 2013). In the spectral domain from UV to the visible, red snow increased rapidly with the wavelength and cryoconite albedo was mostly flat to the wavelength. On the same glacier, *Chlamydomonas nivalis* appeared 94 h after air temperatures remained above freezing, reached a population size of 3.5 × 10^7^ cells · m^−2^, and a growth rate of 0.42 · d^−1^ (Onuma et al. 2018). Using imaging microspectrophotometry on the Greenland ice sheet, intact cells and filaments of *Ancylonema nordenskiöldii*,* Mesotaenium berggrenii*, and *Cylindrocystis brebissonii*, and the cyanobacterium, *Calothrix parietina*, absorb light across UV and PAR, whereas dust particles display little absorption, which suggests that ice algae play an important role in changing albedo that impact melt rates (Yallop et al. 2012). Glacial algae due to their dark pigmentation cause the ice to absorb more solar energy and melt faster (Hodson et al. 2010, Takeuchi 2013, Lutz et al. 2018, Williamson et al. 2019). Simulations indicated that algal blooms influenced snowpack albedo and melt rate using a physical model for the spectral “bioalbedo” of snow (Cook et al. 2017). Their model was used to recreate real spectral albedo data from the High Sierras, California, and broadband albedo data from Mittivakkat Glacier, Greenland. Reduced albedo could be related with Spectral Mixture Analysis (SMA)‐derived snow algae and impurity abundances at albedo levels >45% for algae and >30% for impurities (Huovinen et al. 2018). Red snow in the Arctic plays a key role in decreasing albedo to as much as 13% in one melt season (Lutz et al. 2016, Rossi 2018). The temporal and spatial variations in spectral reflectance on Gulkana Glacier, Alaska Range, Alaska, were due to physical properties and biogenic materials on the glacial surface (red‐colored snow algae and cryoconite; Takeuchi 2009). Surface ablation of the Greenland ice sheet is amplified by darkening caused by light‐absorbing dust, black carbon, and pigmented algae (Stibal et al. 2017). The algal impact was greater than non‐algal impurities yielding a net albedo reduction of 0.038 ± 0.0035 for each algal population doubling. There was significant negative correlation between surface reflectivity and algal biomass or organic matter on a Suntar‐Khayata Mtn glacier in Russian Siberia suggesting that glacial ice algae (*A. nordenskiöldii*) and their products are effective in defining glacial surface albedo, which increased the melting rate 1.6–2.6 times greater than that of impurity free bare ice (Takeuchi et al. 2015).

Photon irradiance and photon fluence rates were measured in snow that contained blooms of *Chlamydomonas nivalis* (Gorton et al. 2001). On a cloudless day, the photon fluence rate at the snow surface was twice that of photon irradiance and was many times greater when the solar angle was low or light was diffuse, and both declined exponentially with depth. At high altitudes (>2,500 m) and polar regions, PAR reflecting off snow may be as high as 5,000 μmol photons · m^−2^ · s^−1^ and UV 30% greater in these snow habitats than compared to sea level, both of which are damaging to phototrophic microorganisms (Morgan‐Kiss et al. 2006). Increased UV‐B irradiance on photosynthesis and pigment composition was measured for 3 days in *Cd. nivalis* from the Austrian Alps without signs of cell damage, but oxygen production was reduced by 20–56% (Remias et al. 2010a). Cells responded by producing more astaxanthin in lipid bodies that surround the chloroplast shielding it from damaging UV‐B. Astaxanthin blocked blue light that was supported by Gorton et al. (2001), and unknown absorbers blocked UV radiation, whereas astaxanthin absorbed UV preventing most of it from reaching the chloroplast, which protected it from excessive damage (Gorton and Vogelmann 2003). Using a hyperspectral microscopic mapping and imaging technique, the red‐colored snow algae*, Cd. nivalis*,* Chlainomonas* sp., and *Chloromonas* sp., collected from the Austrian Alps were studied in the laboratory for photo‐acclimation and protection to high light irradiance (Holzinger et al. 2016). Between 400 and 900 nm, a high absorbance occurred in *Cd. nivalis* and *Chlainomonas* sp. due to secondary carotenoids (astaxanthins), but in the mostly green‐colored *Chloromonas* sp. this high absorbance was missing. To investigate whether cellular water loss influenced spectral properties, cells were plasmolyzed or desiccated, and these treatments had little effect. Higher levels of astaxanthin were found in populations of *Chlainomonas* from Slovakia at the end of the growing season when compared to populations earlier in the growing season from Austria (Procházková et al. 2018b). Both populations were photoinhibited above 1,300 μmol PAR · m^−2^ · s^−1^ compared to *Cd. nivalis* with no photoinhibition up to 2,000 μmol PAR · m^−2^ · s^−1^. Irradiance levels were less than 200 μmol PAR · m^−2^ · s^−1^ in red snow caused by *Ch. kolii* on Mt. Philistine, New Zealand (Novis 2002a). Two snow communities of *Cr. nivalis* in the Czech Republic's Giant Mountains were exposed to a maximum irradiance of 2,000 μmol PAR · m^−2^ · s^−1^ and UVR of 0.135–2.27 mW · cm^−2^ (Kvíderová 2010), and in snow with *Cr. nivalis* in the Carpathians of Slovakia irradiance levels reached 2,133 μmol PAR · m^−2^ · s^−1^ (Hanzelová et al. 2018). In Svalbard incident, irradiance for *Cd. nivalis* in red snow was from 11 to 1500 μmol PAR · m^−2^ · s^−1^ (Stibal et al. 2007). Cell wall components (possibly sporopollenin) exhibited UV absorbance, and UV radiation of wavelengths 280–315 and 315–400 nm dropped to 50% incidence levels in the top 1 and 2 cm, respectively (Gorton and Vogelmann 2003). Zoospores in *Cr. nivalis* revealed acclimatization of the photosynthetic apparatus when photochemical processes remained relatively stable in changing light and UV radiation (Kvíderová 2009). While many snow and glacial algae are exposed at the surface to high VIS and UV irradiance levels, other populations thriving deep under the snow surface cope with low PAR levels to reach the light saturation point, which was the case for *Scotiella cryophila* K‐1 cell types collected from depths of 20–40 cm (Remias et al. 2018). The cells showed photoinhibition at irradiances greater than 70 μmol PAR · m^−2^ · s^−1^. Cysts of the snow alga, *Cr. hindakii*, harvested from high light conditions were photoinhibited at levels above 600 μmol PAR · m^−2^ · s^−1^ (Procházková et al. 2019b). This was three times higher than those from low light conditions, indicating photophysiological adaptation mechanisms.

Using laboratory experiments, long photoperiods, blue light, and low irradiance levels were optimal for sexual reproduction in the snow algae, *Chloromonas tughillensis* (Hoham et al. 2000a,b) and *Cr. chenangoensis* (Hoham et al. 2009), which are so far known only from upstate New York. Both species appear in green snowpacks under tree canopies in April when the photoperiod is 14:10 h light:dark. However, when using photoperiods from 20:4 to 24:0 h light:dark for *Cr. tughillensis* and 24:0 h light:dark for *Cr. chenangoensis*, sexual reproduction increased significantly, which implied the natural habitat was not optimal for sexual reproduction. Optimal PAR levels were 95 for *Cr. tughillensis* and 70–145 μmol PAR · m^−2^ · s^−1^ for *Cr. chenangoensis*.

### Temperature

Many species of snow and glacial algae have a restricted range for growth between 0 and 10°C with optimum temperatures between 0 and 5°C (Table 2), and these may be considered obligate cryophiles or psychrophiles. Other species have broader ranges for growth from 0 to 15, 0 to 20, and −3 to 30°C and may be termed psychrotrophic. Definitions may vary as to what are true psychrophiles and psychrotrophs (Morita 1975, Hoham and Duval 2001, Bölter 2004, Cvetkovska et al. 2017). Since melting snow and ice surfaces have a stable temperature slightly above 0°C, testing the growth optima of individual strains may not relate directly to ecological field conditions. Psychrophilic green algae in the Chlamydomonadales are among the best available models for studying psychrophily and extreme life in photosynthetic eukaryotes (Cvetkovska et al. 2017). The temperature optima for three species causing green snow in upstate New York, *Chloromonas chenangoensis* (2.5–5.0°C), *Cr. rosae* v*. psychrophila* (4–15°C), and *Cr. tughillensis* (2.5–5.0°C), were the first reports from eastern North America (Hoham et al. 2008a). *Chloromonas arctica*, isolated from Svalbard, grew at both temperatures tested in the laboratory, 5 and 20°C, which suggested a greater psychrotolerance for growth (Barcyt≐ et al. 2018). An isolate of *Raphidonema nivale* from Svalbard grew best at 12°C and 200 μmol PAR · m^−2^ · s^−1^ and was considered a soil species only occasionally brought on snow where cells showed great sign of damage (Stibal and Elster 2005). Both factors affected shape and size of cells, the number of cells in filaments, and pleiomorphism.

**Table 2:** Temperature ranges and temperature optima for algae found in snow and glaciers

Species	Location	Temperature range (°C) for growth	Temperature optima (°C)	References
*Chlainomonas kolii*	Olympic Nat. Pk., WA	0–4	0–4	Hoham (1975a)
*Chlainomonas rubra*	Stuart Range, WA	0–4	0–4	Hoham (1975a)
*Chlamydomonas nivalis*	Beartooth Mtns., MT‐WY	−3 to 30	−3 to 20	Mosser et al. (1977)
*Chlamydomonas raudensis*	Antarctica		8	Pocock et al. (2011)
*Chloromonas arctica*	Svalbard (Norway)	5 and 20		Barcyt≐ et al. (2018)
*Chloromonas chenangoensis*	Chenango Valley, NY	0–7.5	2.5–5.0	Hoham et al. (2008a)
*Chloromonas pichinchae*	Stuart Range, WA	0–10	1	Hoham (1975a, 1980)
*Chloromonas polyptera*	Windmill Is., Antarctica	0–10	3	Ling and Seppelt (1998)
*Chloromonas rosae* v. *psychrophila*	Whiteface Mtn., NY	0–20	4–15	Hoham et al. (2008a)
*Chloromonas rosae* v. *psychrophila*	White Mtns., AZ	0–20	4–15	Hoham et al. (2008a)
*Chloromonas rubroleosa*	Windmill Is., Antarctica	0–10	1–4	Ling and Seppelt (1993)
*Chloromonas tughillensis*	Tughill Plateau, NY	0–10	2.5–5.0	Hoham et al. (2008a)
*Chlorosarcina antarctica*	Windmill Is., Antarctica	0–10	3	Ling (1996)
*Chromulina chionophilia*	Mt. Seymour, BC, Canada	0–10	5	Stein (1963)
*Chromulina chionophilia*	Pugh Mtn., WA	0–15–?		Hoham (1975a)
*Cryptomonas frigoris*	High Tatra Mtns. Slovakia	2–10	5?	Javornický and Hindák (1970)
*Cylindrocystis brébissonii*	Pugh Mtn., WA	0–20	10	Hoham (1975a)
*Desmotetra antarctica*	Windmill Is., Antarctica	0–15	2–10	Ling (2001)
*Desmotetra aureospora*	Windmill Is., Antarctica	0–15	2–10	Ling (2001)
*Mesotaenium berggrenii*	Austrian Alps	0–20	1–10	Remias et al. (2009)
*Palmellopsis* sp.	Windmill Is., Antarctica	0–10	3	Ling (1996)
*Raphidonema nivale*	Stuart Range, WA	0–15	5	Hoham (1975a)
*Raphidonema tatrae*	High Tatra Mtns. Slovakia	0–10	4	Hindák and Komárek (1968)

### pH and conductivity

Snow algae are usually found in snow with acidic pH, but there are some exceptions where the pH may be neutral to alkaline (Table 3). The pH optima are known for only a few species from laboratory experiments (Hoham and Duval 2001, Hoham et al. 2007), and in most cases they coincide with pH readings recorded from the field. Only three species have been found in snow with mostly alkaline pH, *Chloromonas chenangoensis* from upstate New York (pH 6.7–7.6) associated with limestone outcrops in shallow snow (Hoham et al. 2007) and *Cr. polyptera* (pH 7.3–8.1) and *Desmotetra antarctica* from polar regions (pH 6.8–7.8) often associated with sea salt sprays and animal wastes (Ling and Seppelt 1998, Ling 2001, Remias et al. 2013b, Lutz et al. 2015b). Conductivity readings from snow are usually low and often not much higher than demineralized water readings usually below 20 μS · cm^−1^ (Table 3). The pH ranged from 3.4 to 4.4 in red snow and averaged 5.4 in green snow in the Slovakian Carpathian Mountains and conductivity ranged from 3.9 to 147 μS · cm^−1^ (Hanzelová et al. 2018). However, in animal rookeries associated with algae in Antarctica, readings may be much higher reaching hundreds of μS · cm^−1^ (Table 3).

**Table 3:** pH (field), pH optima (lab), and meltwater electrical conductivity (EC) for snow and glacial algae

Species	Geographical location	pH (field)	pH optima	EC μS · cm⁻¹ (field)	References
*Ancylonema nordenskiöldii*	Tyndall Glacier, Chile	5.9		2.5	Takeuchi and Kohshima (2004)
*Ancylonema nordenskiöldii*	Svalbard (Norway)	4.7–6.0		3.7–20.1	Remias et al. (2012a)
*Chlainomonas* sp.	Austrian Alps	5.4–5.9		2.6–7.4	Remias et al. (2016)
*Chlainomonas kolii*	Stuart Range, WA	4.9–5.3			Hoham (1974a)
*Chlamydomonas nivalis*	High Tatra Mtns., Poland	5.5			Kawecka (1978)
*Chlamydomonas nivalis*	Svalbard (Norway)	5.0–7.5		5–75	Stibal et al. (2007)
*Chlamydomonas nivalis*	Carpathian Mtns., Slovakia	3.4–4.4			Hanzelová et al. (2018)
*Chlamydomonas nivalis*	Qaanaaq Glacier, Greenland	5.3–6.2		0.4–4.0	Onuma et al. (2018)
*Chloromonas brevispina*	Mt. Rainier Nat. Pk., WA	5.0–5.1			Hoham et al. (1979)
*Chloromonas chenangoensis*	Chenango Valley, NY	6.7–7.6	7.0–8.0	2–8	Hoham et al. (2007)
*Chloromonas hindakii*	Czech Republic, Poland, Slovakia	5.5–7.0		5.1–33	Procházková et al. (2019b)
*Chloromonas hohamii*	AZ, MT, WA mountains	4.7–5.2		4–20	Hoham et al. (1983)
*Chloromonas nivalis*	Mt. Rainier Nat. Pk., WA	5.0–5.1			Hoham and Mullet (1977, 1978)
*Chloromonas nivalis*	Austrian Alps	4.0–6.2		2.8–7.2	Remias et al. (2010b)
*Chloromonas nivalis*	Carpathian Mtns., Slovakia	5.4			Hanzelová et al. (2018)
*Chloromonas nivalis* v. *tatrae*	High Tatra Mtns., Slovakia	5.7		15	Procházková et al. (2018b)
*Chloromonas pichinchae*	Stuart Range, WA	4.9–5.2	5.5–6.5		Hoham (1975b, 1980)
*Chloromonas polyptera*	Windmill Is., Antarctica	6.7–8.1		56–950	Ling and Seppelt (1998)
*Chloromonas polyptera*	Antarctic Peninsula	7.4–7.5		42–95	Remias et al. (2013b)
*Chloromonas polyptera*	Iceland	7.7–7.9			Lutz et al. (2015a)
*Chloromonas reticulata*	Ural Mtns., Russia	5.3–6.4		6.3–8.8	Novakovskaya et al. (2018)
*Chloromonas rosae*	Whiteface Mtn., NY	4.9–5.2	4.0–5.0	4–15	Hoham and Duval (2001)
v. *psychrophila*	White Mtns., AZ	4.9	4.5–5.0	4–15	
*Chloromonas rubroleosa*	Windmill Is., Antarctica	4.6–6.2		25–85	Ling and Seppelt (1993)
*Chloromonas tughillensis*	Tughill Plateau, NY	5.0–5.3	4.9–6.3	6–17	Hoham et al. (2007)
*Chlorosarcina antarctica*	Windmill Is., Antarctica	6.3–6.9		39–44	Ling (2002)
*Desmotetra antarctica*	Windmill Is., Antarctica	6.8–7.8		279–426	Ling (2001)
*Hydrurus* sp.	King George Is., AntarctSvalbard (Norway)	5.75.9		26.53.8	Remias et al. (2013a)
*Mesotaenium berggrenii*	Windmill Is., Antarctica	4.5–5.7		6–33	Ling and Seppelt (1990)
*Sanguina aurantia*	Svalbard (Norway)	4.7–7.1		3–26–(84)	Procházková et al. (2019a)
*Sanguina nivaloides*	World wide	4.7–7.1		3–26–(84)	Procházková et al. (2019a)
*Scotiella cryophila* K‐1	Austrian Alps	5.1–5.5		7–9	Remias et al. (2018)

### Nutrients

At two sites in the Giant Mtns., Czech Republic, the chemical composition did not differ significantly except for increased total phosphorus in one site where cysts of *Chloromonas brevispina* and *Cr. nivalis* dominated and increased total nitrogen in the second site where only zoospores of *Cr. nivalis* were found (Kvíderová and Kociánová 2011). Red snow caused by *Chlainomonas kolii* on Mt. Philistine, New Zealand, decreased levels of NH_4_‐N to 1.1 μg · L^−1^, which was the only nutrient influenced by the growth of the algal population (Novis 2002a). Microbial communities (algae, bacteria, and archaea) on Icelandic glaciers were not nutrient limited due to nutrient rich and fast dissolving volcanic ash (Lutz et al. 2015a). Most snow algal communities examined from glaciers on stratovolcanoes in the Pacific NW, USA, were not limited by phosphorus or fixed nitrogen, sequestered Fe, Mn, and P leached from minerals from local rocks, and it was suggested that DIC may be the limiting nutrient (Hamilton and Havig 2017). In the same habitat, snow algae drive light‐dependent carbon uptake that is supported by fixed nitrogen from deposition from precipitation (Havig and Hamilton 2019). This highlights intense cycling of carbon and nitrogen that is driven by supraglacial microbial communities that feed subglacial microbial communities. Production of metabolites in snow and glacial algae in Svalbard and Arctic Sweden was driven mainly by nitrogen and less so by phosphorus limitation (Lutz et al. 2017). This is important for the synthesis of secondary carotenoids, which cause a darkening of glacial surfaces that lead to decreased albedo and higher melting rates. Eight snow algal communities from Svalbard were analyzed for nutrient concentrations and fatty acid composition that revealed a range of NH_4_^+^ (<0.005–1.2 mg N · L^−1^), PO_4_^−3^ (<18 μg · L^−1^), and FA (50–300 mg FA · g C^−1^; Spijkerman et al. 2012). Differences in red, green, and orange color and nutritional composition between patches of snow algal communities within one snowfield were not directly related to nutrient conditions, but perhaps from parameters such as slope, meltwater rivulets, and rock formation. On Livingston Island, Antarctica, coastal snowpacks fertilized by greater nutrients from rock debris and maritime fauna developed pigmented snow algal communities far greater than inland glacial snowpacks (Hodson et al. 2017). The snow chemical composition in two snow communities of *Cr. nivalis* in the Czech Republic's Giant Mountains was similar at both sites regardless of whether snow algae were present (Kvíderová 2010). Only the concentration of P‐PO_4_^−3^ was significantly higher in the presence of algae. In the Carpathians of Slovakia, levels of N‐NO_3_ were greater in snow with *Cr. nivalis* than N‐NH_4_ or P‐PO_4_ (Hanzelová et al. 2018).

Extracts from balsam fir leaf litter collected from Whiteface Mtn., Adirondacks, NY, enhanced the growth of the snow alga, *Chloromonas rosae* v*. psychrophila* (Hoham et al. 2008b). This was the only snow alga found in these green snowbanks under this conifer, and unidentified leachates from these fir needles appear to be a selective advantage. Bark and leaf litter extracts from five species of conifers above green snowbanks in the Stuart Range of western Washington showed that growth in the snow alga, *Cr. pichinchae*, was mostly enhanced and that of *Raphidonema nivale* was often suppressed (Hoham and Duval 2001). These results support the conclusion that litter from these conifers was influencing the high populations of the former and low populations of the latter in this green snow.

Fe, a key micronutrient for photosynthetic growth, is necessary to support the formation of high‐density snow algal blooms (Harrold et al. 2018). Using Fe^90^ in *Chloromonas brevispina*‐bacterial coculture experiments, snow algal growth was stimulated. There was a decrease in the ratio of bacteria (Gammaproteobacteria, Betaproteobacteria, and Sphingobacteria identified using 16S rRNA analyses) to algae compared to those of Fe‐depleted conditions. The dominance of Betaproteobacteria in snow with *Chloromonas* suggested that these bacteria can utilize available carbon in algal‐rich habitats and may promote algal growth (Terashima et al. 2017).

## Biotechnology and commercial uses

Polar algae (snow/soil) from Svalbard cultivated at 6°C produced high cell densities and productivity that yielded primarily C16 and C18 PUFAs, which implied excellent opportunities for producing food and fuel products (Hulatt et al. 2017). Snow species in *Chloromonas* were found to be a major producer of C16 PUFAs (16:3 and 16:4) and as a potential biotechnological source of them (Řezanka et al. 2014). Under stress conditions of high light and low nitrogen levels, 10 algal strains from snow and permafrost synthesized elevated amounts of secondary carotenoids (8 Chlorophyceae) or α‐tocopherol (2 Trebouxiophyceae; Leya et al. 2009). It was suggested that some of these strains might be candidates for biotech applications. Other examples may include products from psychrophilic green algae, *Chlamydomonas nivalis* (astaxanthin), *Chloromonas* sp. (glycerol), *Mesotaenium berggrenii* (sucrose, glucose, glycerol), and *Raphidonema* sp. (α‐tocopherol and xanthophylls) and from cyanobacteria (myxoxanthophylls and canthaxanthin) (Varshney et al. 2015). Since cryophilic species of *Sanguina*,* Ancylonema*, and *Mesotaenium* have not been cultured successfully, only field samples of these species can be employed for biotech and commercial uses at this time.

## Conclusions

Snow and glacial algae are unique organisms that live in one of the most extreme environments on Earth. Research has included their geographic distribution, community structure, life cycles and reproductive strategies, primary productivity, secondary metabolites, genomics, systematics, evolution, environmental factors (light, temperature, pH, conductivity, and nutrients), and biotechnology. Satellite imagery has increased our understanding of their distributions and abundance. Community structure and complex life cycles will be enhanced through combined field with laboratory studies, which should help resolve the problem of acquiring strains of *Sanguina* and *Ancylonema*. With more metagenomic, transcriptomic, multigene, and ITS analyses, species differentiation and biodiversity will become clearer. A major current problem for reliable molecular community studies using HTS in snow and glacial algae appears to be the low resolution of the 18S rDNA marker at the species level. New and improved molecular phylogenies will further our knowledge of the evolutionary histories of these microbes, and more species outside the Chlamydomonadales (Chlorophyta) and Zygnematales (Streptophyta) need to be examined. This should include tropical glaciers and their communities because these habitats are most threatened globally. Snow and glacial algae are having profound effects on the melting of snow and glaciers. As climate change continues along with warmer global temperatures, these algae will have further impacts on the melting process. The maximum irradiance limits that snow and glacial algae can tolerate in open exposures need more investigations as more is known about low irradiance tolerances in species living under tree canopies or deep in snow. More studies of secondary metabolites will help to better understand protection from potentially damaging UV and PAR. With additional studies worldwide, there appears to be a wider tolerance to pH than realized, but most species are found in acidic snow or ice. Many species are cold adapted not growing above 10°C and appear restricted to their cryophilic surroundings. Further research into IBPs and PUFAs should reveal more information on survival in subfreezing temperatures. Some species are found in areas of high nutrients or conductivity, and these are associated with animal rookeries in polar regions. However, most species of snow and glacial algae are found in low conductivities with more limited nutrients. More work is needed to better understand how these algae along with other community members are contributing and interacting in the nutrient and water cycles in snow and ice. Strains of these algae have potential in commercial products for food or fuels, and this area has barely been explored.

We thank the Austrian Science Fund (FWF) project P29959 to DR for funding. We also thank both reviewers for their constructive comments, and RH thanks Dr. Pete Siver, Connecticut College, for his encouragement to do this Review.
